# Primary care physicians play a crucial role in diagnosing and managing rare eosinophilic diseases: HES and EGPA

**DOI:** 10.3389/fmed.2025.1568770

**Published:** 2025-06-24

**Authors:** Mili Shum, Ora Gewurz-Singer, Jared Silver, Praveen Akuthota

**Affiliations:** ^1^Department of Dermatology, Section of Allergy and Immunology, University of Utah Health, Salt Lake City, UT, United States; ^2^Division of Rheumatology, Department of Internal Medicine, University of Michigan, Ann Arbor, MI, United States; ^3^Medical Affairs, GSK, Research Triangle Park, Durham, NC, United States; ^4^Division of Pulmonary, Critical Care, Sleep Medicine & Physiology, Department of Medicine, University of California, San Diego, San Diego, CA, United States

**Keywords:** hypereosinophilic syndrome, eosinophilic granulomatosis with polyangiitis, Churg–Strauss, eosinophils, primary care

## Abstract

Hypereosinophilic syndrome (HES) and eosinophilic granulomatosis with polyangiitis (EGPA) are chronic eosinophilic diseases with serious multisystem manifestations. Patients with HES or EGPA often fail to receive a timely diagnosis, and while these conditions are considered rare, frequent under-recognition indicates that their true prevalence likely exceeds current estimates. Increased primary care physician (PCP) awareness of these systemic eosinophilic conditions and the “red flags” that should trigger referral will help more patients receive timely diagnosis and care. Patients with HES or EGPA present with a heterogeneous range of symptoms and manifestations that can overlap with other conditions, making diagnosis challenging. PCPs should be aware that the following are red flags that warrant further investigation and trigger expert referral: blood eosinophil count ≥10% of total peripheral white blood cells or ≥1,000 cells/μL; persistent hypereosinophilia, noting that systemic corticosteroid treatment may variably impact the degree of eosinophilia; refractory asthma symptoms with the need for prolonged or recurrent systemic corticosteroid treatment; reports of decreasing efficacy to asthma therapy; extra-pulmonary findings in the setting of eosinophilia; multiorgan system involvement; and evolving or worsening signs and symptoms over periods of weeks to months or years. PCPs play a key role in the diagnosis and management of rare eosinophilic diseases. By being aware of HES and EGPA and their associated red flags, PCPs are well-placed to recognize these conditions early, trigger further investigations, and coordinate effective multidisciplinary care. This can help patients receive a more accurate diagnosis on time and faster access to treatment, which may ultimately improve patient outcomes.

## 1 Introduction

Eosinophilic diseases are a group of heterogeneous inflammatory disorders that have blood and/or tissue eosinophilia as a common feature and are driven by mechanisms, including local and systemic overproduction of eosinophilic cytokines (1–3). Many eosinophilic diseases are rare and under-recognized; the significance of symptoms resulting from eosinophilic inflammation is overlooked in clinical practice (4, 5). Two serious multisystem conditions with complex diagnostic challenges are hypereosinophilic syndrome (HES) and eosinophilic granulomatosis with polyangiitis (EGPA) (Box 1) (5, 6).

A variety of manifestations and changing presentation as the conditions evolve, combined with unclear diagnostic guidance, complex referral pathways, and a paucity of disease awareness, contribute to delayed diagnosis, sometimes by months or years. Symptoms are often managed with high-dose oral corticosteroids (OCSs), which could be associated with cumulative dose-related toxicity (3, 7). Longer waits in accessing targeted, less toxic treatment can increase the disease burden for patients and could increase the opportunity for potentially irreversible end-organ damage (5–7). Primary care physicians (PCPs) play critical roles in delivering effective care to patients with suspected HES/EGPA by recognizing signs and symptoms during early and repeated patient interactions and coordinating referrals to appropriate specialists (6).

Currently, there are classification criteria for EGPA, but no universally accepted diagnostic criteria. A recent publication (2024) provides physicians with the first consensus checklist of clinical “red flags” to raise suspicion of EGPA, to encourage further testing, and to support earlier detection and improve EGPA management (8, 9). To date, no comprehensive evaluation of these “red flags” to support diagnosis of both HES/EGPA exists. Using a case study, this article aims to highlight the importance of early intervention in HES/EGPA and share “red flags” and “pearls of wisdom” aimed at PCPs, to guide them on the steps leading to diagnosis. This information is critical for PCPs striving to improve the patient journey, ensuring potentially organ-/life-threatening diagnoses are not delayed and excessive disease burden is avoided (6). While the rarity of HES/EGPA presents challenges for disease management, PCPs have the support of their multidisciplinary network, which they must coordinate to allow timely intervention with precision medicine (10, 11).

## 2 Example case study

**Figure 1:**
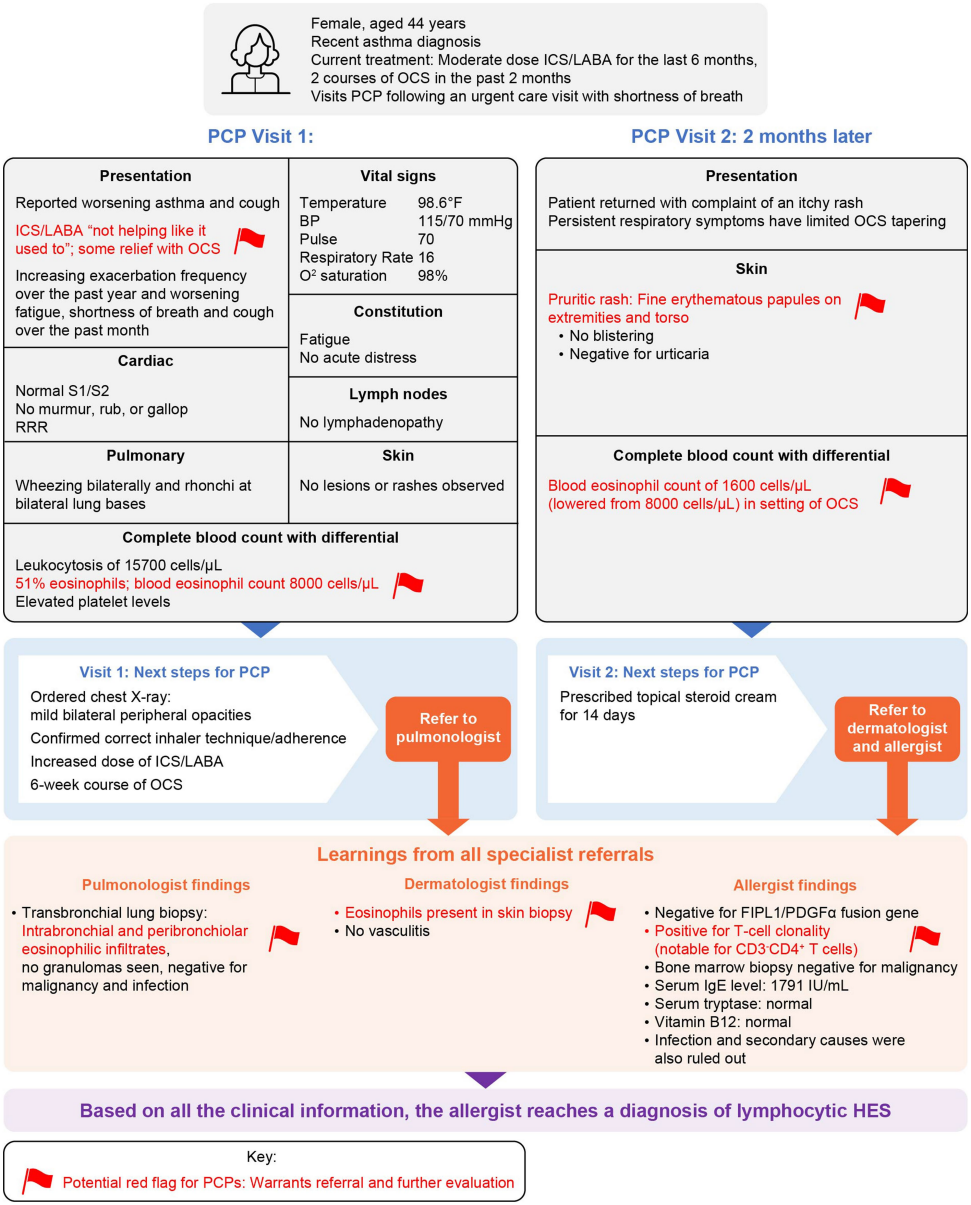
Case study: Patient told to meet a PCP following an urgent care visit. BP, blood pressure; BEC, blood eosinophil count; FIPL1/PDGFα, Fip1-like-1-platelet-derived growth factor receptor alpha; HES, hypereosinophilic syndrome; ICS, inhaled corticosteroid; IgE, immunoglobulin E; LABA, long-acting β_2_-agonist; OCSs, oral corticosteroids; PCP, primary care physician; RRR, regular rate and rhythm.

A 44-year-old woman with new-onset asthma presented at urgent care with shortness of breath and was treated with an OCS course before following up with a PCP (Figure 1). On the first PCP visit, the patient reported an increased frequency of asthma exacerbations in the last year, resulting in the prescription of two OCS courses in the past 2 months, and worsening fatigue, shortness of breath, and cough over the past month. The patient detailed that her symptoms had worsened despite using a moderate-dose inhaled corticosteroid and long-acting β_2_-agonist (ICS/LABA) for the last 6 months. The patient relayed that ICS/LABA medication was “not helping like it used to”; however, OCSs provided some relief. Physical examination revealed bilateral wheezing and rhonchi at the bilateral lung bases. Blood tests revealed an elevated leukocyte count with 51% eosinophils and blood eosinophil count of 8,000 cells/μL. Prior medical, smoking, and travel history did not raise any alarms for infection or malignancy. The PCP ordered a chest X-ray, which showed faint bilateral peripheral opacities. The patient was prescribed an increased ICS/LABA dose with a 6-week OCS course and referred to a pulmonologist.

*Red flag*: Patient reported decreasing efficacy of ICS/LABA and/or OCSs.
*Red flag*: Blood eosinophil counts of 8,000 cells/μL exceeding the threshold for hypereosinophilia (≥1,500 cells/μL).

At a follow-up visit 2 months later, the patient returned with a new-onset rash. On examination, the skin had fine pruritic erythematous papules distributed across the extremities and torso, without blistering. Blood tests revealed continued hypereosinophilia, with blood eosinophil counts of 1,600 cells/μL, although this was a substantial reduction from previous levels after the OCS treatment she had received. The PCP prescribed topical steroid cream for 14 days and referred the patient to a dermatologist and allergist.

*Red flag*: Hypereosinophilia remained despite recent treatment with corticosteroids.
*Red flag*: Involvement of another organ system, with rash in addition to asthma.

Transbronchial lung and skin biopsies showed eosinophilic infiltrates and no vasculitis. Absence of vasculitis in a biopsy can be related to the quality of the biopsy, the site chosen, the disease stage, or the true absence indicative of HES. This evidence of eosinophilia-attributable damage and/or dysfunction, together with blood eosinophil counts ≥1,500 cells/μL on two examinations and the exclusion of other disorders, met the criteria for HES (1, 2, 4, 12). Based on the blood eosinophil count (≥1,000 cells/μL) and asthma history while on ICS/LABA, EGPA was also suspected (8, 13); however, clinical workup revealed HES. The presence of pulmonary and cutaneous manifestations identified a multisystem disorder, rather than organ-restricted HES. Thorough diagnostic workup from the allergist ruled out infections and secondary causes of eosinophilia and revealed CD3^−^ CD4^+^ T-cell clonality, typical of lymphocytic HES (L-HES). Based on the collective clinical information from the specialist and PCP visits, the allergist diagnosed L-HES.

As a next step, relevant specialists initiated a comprehensive assessment of major organ systems. Since L-HES can transition to cancer, the PCP referred the patient to hematology for monitoring and further diagnostic workup, as needed. Moving forward, the continued identification of new patient complaints by the PCP can help inform ongoing care. PCPs tend to know their patients well and are well placed to detect HES exacerbations and flares early. Furthermore, they can provide age-appropriate screenings (e.g., cancer) and monitor for common health conditions (e.g., diabetes, hypertension, and hyperlipidemia), of particular importance with corticosteroid exposure.

## 3 Information and guidance for PCPs

### 3.1 Functions of eosinophils and relevance in clinical practice

Eosinophils are granulocytes with inflammatory and immunomodulatory roles (14). While eosinophils contribute to healthy tissue homeostasis and fighting against infection, they also play pathologic roles in diseases including asthma, chronic rhinosinusitis with nasal polyps, gastrointestinal (GI) disorders, HES, and EGPA (3, 15, 16). A blood eosinophil count of >500 cells/μL is considered to be elevated, while a count of ≥1,500 cells/μL indicates hypereosinophilia (1, 4, 12). In certain clinical settings, high eosinophil levels and inappropriate activation can lead to organ and tissue damage, both through direct cytotoxicity and indirectly via the mediation of other inflammatory cells (3, 15, 16). Eosinophil-activating cytokines (e.g., interleukin-5) are key drivers of eosinophil development, regulation, and pathogenesis (14, 16). In primary care settings, tests routinely used for symptom investigation or monitoring of existing conditions include a complete metabolic panel and a complete blood count (CBC) with differential (17). The CBC with differential includes a description of individual lymphocytic components and other white blood cell (WBC) lineages, beyond the total WBC count, that may not be considered during routine clinical care. However, the eosinophil count can contain critical clinical information and should be routinely reviewed.

### 3.2 Key features of HES and EGPA

**Figure 2:**
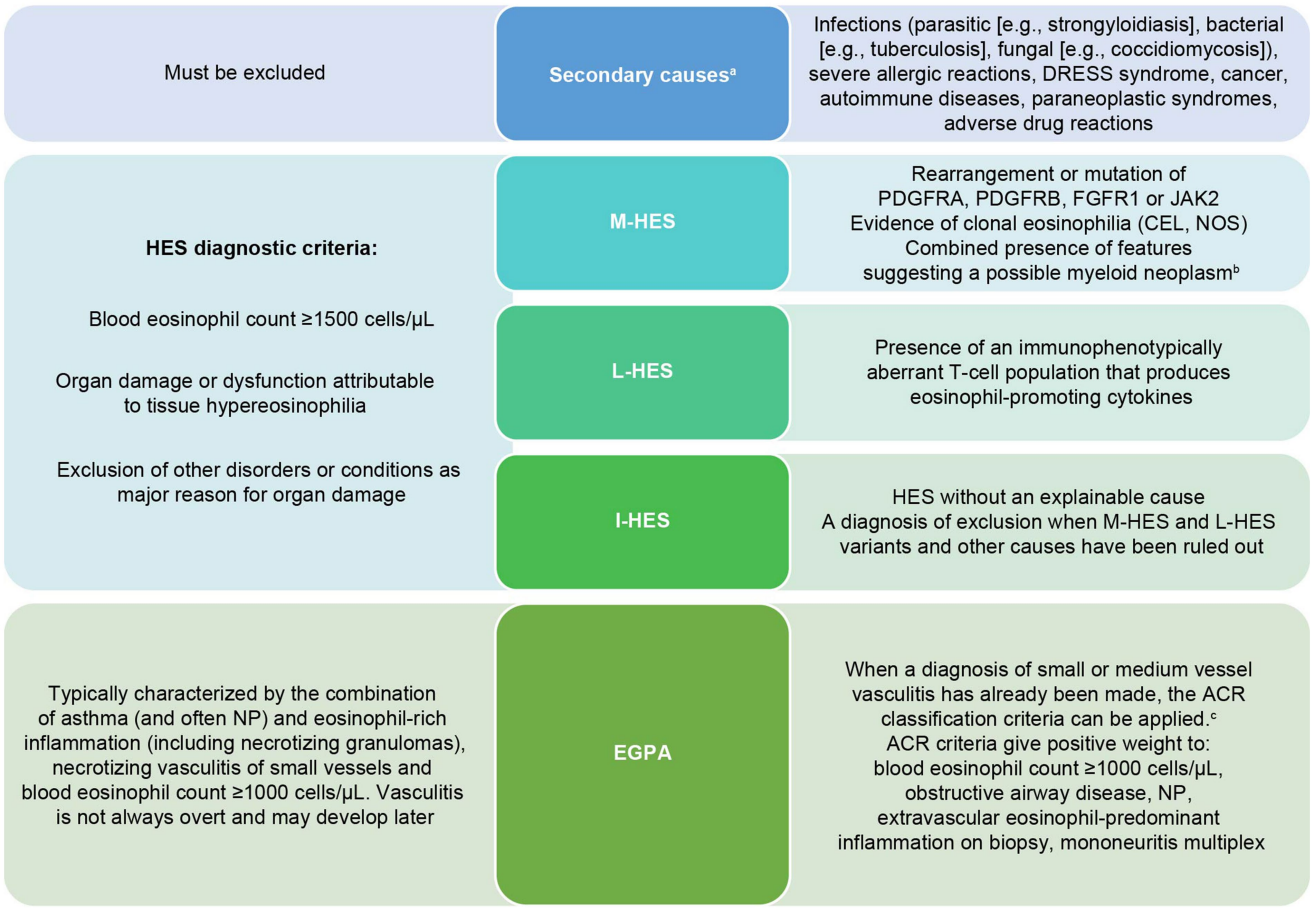
Differential diagnosis of HES and EGPA (1, 4, 13). ^a^Diagnosis or exclusion of some secondary causes may also lead to referrals to a specialist or multidisciplinary team. ^b^Anemia/thrombocytopenia, splenomegaly, elevated serum vitamin B12/tryptase, or lack of response to systemic corticosteroid therapy. ^c^Current ACR/EULAR EGPA classification criteria require a total score of ≥6 across the following seven criteria: obstructive airway disease (+3), NPs (+3), mononeuritis multiplex (+1), blood eosinophil counts ≥1,000 cells/μL (+5), extravascular eosinophilic predominant inflammation on biopsy (+2), positive test for cytoplasmic ANCAs or anti-PR3 antibodies (−3), and hematuria (−1) (13). ACR, American College of Rheumatology; ANCAs, antineutrophil cytoplasmic antibodies; CEL, chronic eosinophilic leukemia; NOS, not otherwise specified; DRESS, Drug Reaction with Eosinophilia and Systemic Symptom (DRESS); EGPA, eosinophilic granulomatosis with polyangiitis; EULAR, European Alliance of Associations for Rheumatology; FGFR1, fibroblast growth factor receptor 1; I-HES, idiosyncratic hypereosinophilic syndrome; JAK2, Janus kinase 2; L-HES, lymphocytic-variant hypereosinophilic syndrome; M-HES, myeloid hypereosinophilic syndrome; NP, nasal polyps; PDGFRA, platelet-derive d growth factor receptor alpha; PDGFRB, platelet-derive d growth factor receptor beta; PR3, proteinase 3.

**Figure 3:**
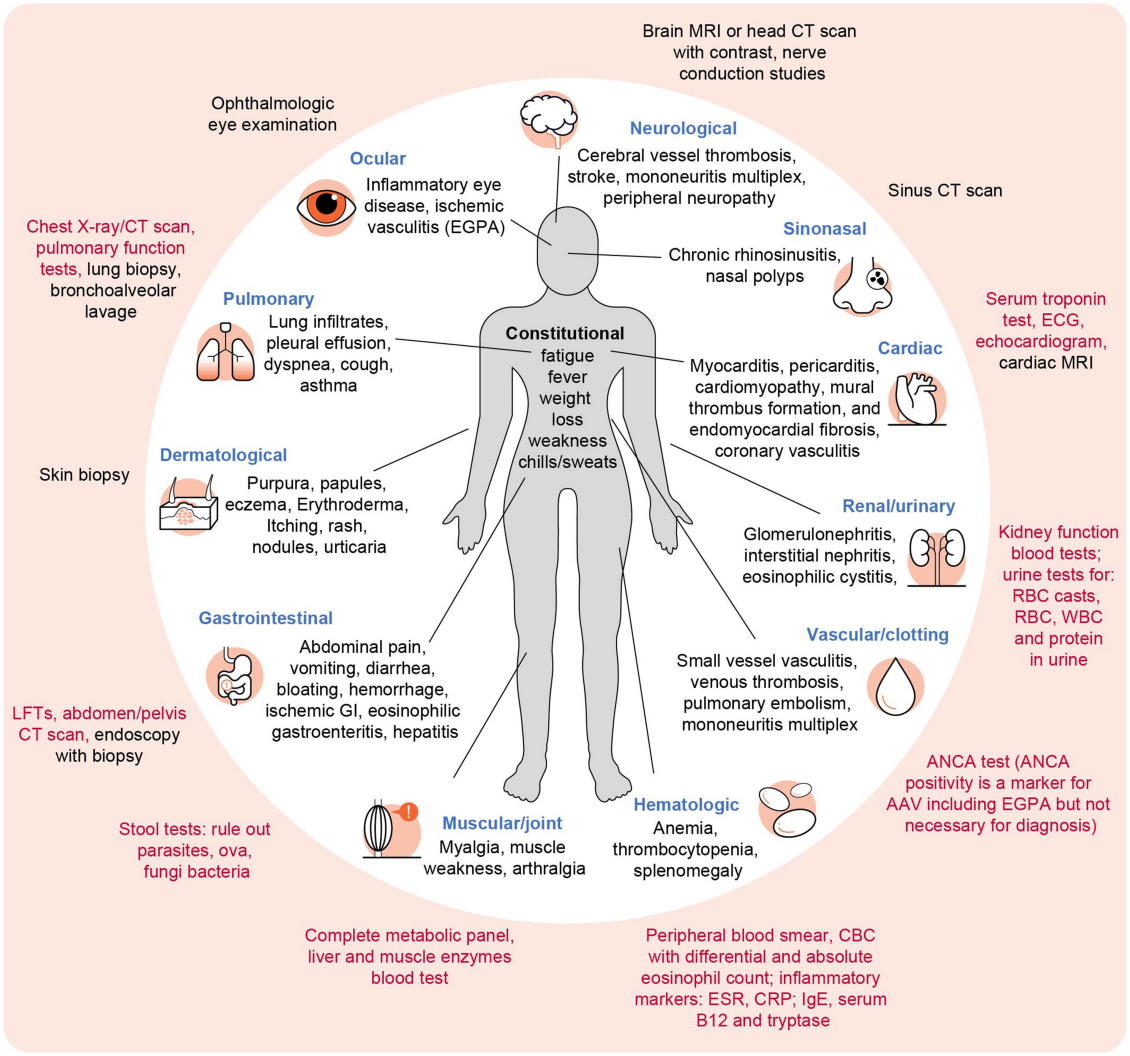
Potential systems and symptoms involved in HES and EGPA and associated diagnostic workup (2, 4, 5). Inner circle: symptoms and manifestations in HES and EGPA. Outer circle: diagnostic tests and workup performed throughout diagnosis (or for monitoring, post-diagnosis); tests that the PCP is likely to initiate are shown in red, while tests more likely to be performed following a specialist referral are shown in black. AAV, ANCA-associated vasculitis; ANCAs, antineutrophil cytoplasmic antibodies; CBC, complete blood count; CMP, complete metabolic panel (includes renal function, electrolytes, and liver function); CRP, C-reactive protein; CT, computed tomography; EGPA, eosinophilic granulomatosis with polyangiitis; ECG, electrocardiogram; ESR, erythrocyte sedimentation rate; GI, gastrointestinal; HES, hypereosinophilic syndrome; IgE, immunoglobulin E; LFT, liver function test; MRI, magnetic resonance imaging; PCP, primary care physician; RBCs, red blood cells; WBCs, white blood cells.

HES and EGPA are rare systemic disorders characterized by heterogeneous manifestations affecting a variety of organs. Their pathophysiology and etiology vary between HES subtypes and EGPA (Figure 2) (2, 5). In addition to constitutional symptoms including fatigue and fever, these symptoms commonly affect the lungs, eyes, sinus, skin, nerves, and GI tract (2, 5). The frequency of each manifestation varies by disease type (e.g., sinus and lung involvement are more common in EGPA, and skin involvement is most common in L-HES) (4). Symptoms and signs depend on the specific organ(s) involved (Figure 3) and may evolve over time (18, 19). Typical patterns of disease development and tempo also vary by disease type; EGPA classically develops in three partially overlapping stages, starting with the prodromal phase (late onset asthma), followed by eosinophilia with eosinophilic tissue infiltration and damage, and finally systemic vasculitis; however, some stages may overlap or not occur at all (20). While asthma is a defining feature of EGPA, many patients with EGPA do not follow the classic pattern of disease evolution. For both HES and EGPA, symptoms and eosinophilia may be persistent or follow a relapsing/remitting disease course, so continual monitoring is important to optimize care (2, 21).

BOX 1 **What are HES and EGPA?**

- HES and EGPA are rare but under-recognized diseases with heterogeneous clinical presentations (6).
  - Estimated annual prevalence: HES: between 1.5 and 8.9 cases per million people (22); EGPA: 15 cases per million adults (23).
- HES refers to a group of disorders diagnosed based on persistent blood hypereosinophilia (≥1,500 cells/μL) and organ damage or dysfunction attributed to tissue hypereosinophilia (2, 4).
  - Main subtypes: myeloid (M-HES), lymphocytic (L-HES), and idiopathic (I-HES) (Figure 2) (5).
- EGPA can share many of the features of HES (4, 5) but can be diagnosed at a lower blood eosinophil count threshold of ≥1,000 cells/μL versus ≥1,500 cells/μL for HES (1, 12, 13). In addition to blood eosinophilia, patients with EGPA typically have asthma and may have nasal polyps and/or evidence of vasculitis such as glomerulonephritis of the kidneys or vasculitic neuropathy (13, 24). Moreover, 30–47% may test positive for antineutrophil cytoplasmic antibodies (ANCAs), although ANCA positivity is not a prerequisite to diagnose EGPA (Figure 2) (4).
- Both HES and EGPA progress over time. Recognizing indicators of prodromal EGPA (late onset asthma and eosinophilia) and initiating treatment early may prevent progression to full vasculitic disease (5).

### 3.3 Role of PCPs in aiding diagnosis and managing HES and EGPA

Combining a thorough assessment of the patient’s history, physical examination, and workup is key to identifying the heterogeneous symptoms indicative of HES/EGPA (Figure 3). It is essential that PCPs are aware of the red flags (Box 2) that warrant further investigation and referral. Once the patient is diagnosed, PCPs will work with their consultants to monitor disease activity. Due to their complex and systemic nature, HES and EGPA are ideally managed by a multidisciplinary team (MDT) (25), with primary management most commonly within hematology, rheumatology, internal medicine, pulmonology, or allergy/immunology (26, 27). The role of the PCP within the MDT may vary depending on factors such as disease activity, distance to specialist centers, frequency of visits to MDT specialists, and local healthcare delivery structures. A key component of PCP involvement is recognizing flares in known disease manifestations and identifying new ones. While overall management responsibility is typically led by a specialist within the MDT with expertise in management of the rare condition, a PCP with regular patient contact is in an excellent position to identify signs that may detect worsening of symptoms of the rare disease and promptly alert the MDT.

Respiratory involvement is common in both HES and EGPA, although asthma and sinus involvement tend to favor EGPA over HES. Asthma symptoms tend to be severe and refractory, requiring OCSs. Patients with EGPA can occasionally present with alveolar hemorrhage and hemoptysis, which warrant urgent referral to emergency care. Skin involvement is also common [e.g., vasculitic rash (purpura), urticaria, or nodules], particularly in L-HES. GI involvement may occur and can overlap with organ-restricted eosinophilic syndromes such as eosinophilic gastritis. Neurologic involvement, including neurologic deficits (mononeuritis multiplex) and neuropathic symptoms (numbness and tingling), may manifest. Life-threatening cardiac and thromboembolic manifestations can develop; therefore, PCPs should check for signs of cardiovascular involvement, including chest pain, shortness of breath, lower extremity swelling, and heart murmurs or irregular rhythms. Renal involvement in EGPA should be suspected in patients with new hypertension, swelling, reduction of glomerular filtration rate, and abnormal urinalysis with protein and/or blood. Where any neurological or cardiac involvement is suspected, prompt referral or emergency evaluation is essential, that is, early intervention could prevent irreversible damage, serious complications, or fatal outcomes. PCPs should be aware of the wide array of symptoms indicative of HES/EGPA and remain vigilant for other organ involvement because any organ system can be impacted.

Regarding diagnosis, an elevated blood eosinophil count is a hallmark of HES/EGPA. A blood eosinophil count of ≥1,500 cells/μL indicate hypereosinophilia, while EGPA has a lower diagnostic threshold: a blood eosinophil count of ≥1,000 cells/μL (1, 4, 12, 13). Finding these eosinophil levels should prompt additional workup to rule out secondary causes of eosinophilia, checking for signs of organ involvement and triggering referral. Owing to the heterogeneity within HES/EGPA presentation and variation in the underlying causes of their subtypes, it is essential to note that not all patients have all symptoms. Diagnosis of HES/EGPA should not be ruled out by a negative test for one particular aspect of the disease. HES and EGPA are progressive, so recognizing new organ involvement early could prevent damage (5).

*Pearl of wisdom: Eosinophil levels may be reduced to normal by steroid treatment. Check eosinophil levels before a prednisone burst is started for a flare of rash/respiratory symptoms. Historical results may also be useful to determine typical eosinophil levels for the individual patient. Blood eosinophil counts ≥1,000 cells/μL should trigger additional workup and referral. Repeated eosinophil levels may be needed to identify hypereosinophilia*.

Potential manifestations of HES/EGPA, with associated laboratory tests and physical examination findings, are summarized in Figure 3. The workup of eosinophilia includes checking for infectious diseases; moreover, in the cause of eosinophilia, stool was tested to evaluate for parasites, ova, or bacteria, and serum blood tests for locally endemic parasites (28, 29). Elevated serum B12 and tryptase levels, tests typically ordered by hematologists and allergists, suggest M-HES (30). Elevated inflammatory markers, including erythrocyte sedimentation rate and C-reactive protein, are non-specific but might be a predictor of flare. While the evidence is not robust, they may have clinical utility in day-to-day practice. Troponin level checks and an electrocardiogram could be obtained to screen for cardiac involvement when there is hypereosinophilia. An increase in blood pressure, a drop-in glomerular filtration rate, presence of red blood cells (RBCs), RBC casts, WBCs, and protein in urine can indicate kidney involvement and should elicit a referral to nephrology. PCPs can support specialists by ordering these tests while awaiting a referral.

*Pearl of wisdom: Although cardiac manifestations are not the most common HES/EGPA manifestation, they are an important cause of morbidity and mortality. Thorough cardiac workup and monitoring are essential in cases of suspected HES/EGPA*.

Finally, although EGPA is classified as ANCA-associated vasculitis (AAV), only ~40% of patients with EGPA test ANCA-positive, with the presence of anti-myeloperoxidase antibodies being more common than anti-proteinase 3 antibodies in serum (24). While a positive ANCA test is indicative of AAV, its absence does not preclude the EGPA diagnosis (13, 24).

A thorough workup is essential to ensure critical organ involvement has not been missed and to identify the role of eosinophils in patient symptoms. Tests that may be completed by specialists after referral are shown in Figure 3 and include the following: electrocardiograms, echocardiograms, or cardiac magnetic resonance imaging for cardiac involvement; computed tomography of the abdomen or pelvis where there is GI involvement; and high-resolution computed tomography pulmonary imaging. Findings from specialist tests that would support an EGPA diagnosis include evidence of vasculitis through biopsy or proxies, i.e., mononeuritis multiplex or glomerulonephritis, which may require referral to a rheumatologist for immunomodulation therapies. Findings suggestive of specific HES subtypes include rearrangement or mutation of the genes *PDGFRA*, *PDGFRB*, *FGFR1,* or J*AK2,* or evidence of clonal eosinophilia (M-HES subtype) and clonal T-cell population that produces eosinophil-promoting cytokines (L-HES subtype).

BOX 2 **Red flags suggesting potential eosinophilic disorders should trigger referral and further investigation**

Red flags that may be noticed by PCPs:

- High percentage or absolute blood eosinophil levels (≥10% of total peripheral WBCs or ≥1,000 cells/μL).
- Persistent hypereosinophilia after corticosteroid treatment. In most cases, OCSs may mask eosinophilia by dampening type 2 inflammation/suppression of eosinophils.
- Need for prolonged or recurrent systemic corticosteroid treatment.
- Reduced responsiveness to asthma treatments.
- Extra-pulmonary findings in the setting of eosinophilia.
- Multiorgan system involvement.
- Abnormal spirometry findings.
- Eosinophilic tissue infiltrates in the lungs, skin, or GI tract (where biopsy is indicated).

### 3.4 Role of PCPs in shortening patient journeys

Prior studies have shown that most patients with HES (70%) visited ≥4 healthcare providers before being diagnosed (31), and for patients with EGPA, most (87%) were likely to visit ≥1 specialist before diagnosis (32). Patients also often experience multiple emergency room and hospital admissions before HES/EGPA diagnosis and proper management. Being an early and repeated point of contact, PCPs have the power to shorten the patient journey. For patients newly presenting with asthma symptoms or aberrant eosinophil counts, PCPs should check for red flags before calling for specialist referral to support timely and accurate diagnosis. For patients already diagnosed with HES/EGPA, ongoing, lifelong multi-specialist and PCP care is required to achieve and maintain remission, monitor disease status, mitigate against end-organ damage and relapse, and minimize medication toxicity. In addition to monitoring patients and identifying new disease manifestations, PCPs also facilitate broader care, including the correct vaccinations, age-appropriate cancer and osteoporosis screenings, and wellness checks (e.g., for mood disorders or nutritional deficits) (33), which places them on the front line for initiating the next steps if signs of disease progression appear.

## 4 Discussion

Patients with HES/EGPA often face a long path to diagnosis and effective treatment, involving multiple contacts with PCPs (6, 31). Early identification and appropriate treatment of progressive eosinophilic disease may improve patient outcomes (2, 7, 34–36). Since PCP actions are critical to expedite the patient journey, PCPs must remain vigilant for disease red flags and make necessary specialist referrals, leading to the comprehensive assessments needed for diagnosis. The “Red flags” described in this study for EGPA broadly align with those of a previously published article, which also highlights the potential for current therapies to dampen eosinophil levels (8), and this should not be overlooked when considering a diagnosis of EGPA.

When managing patients with HES and those with EGPA, it is important to involve a multidisciplinary team with awareness of rare diseases (25); PCPs play a crucial role in the continual monitoring of patients at follow-ups, being well-placed to identify new manifestations in these chronic and evolving diseases, and initiate timely specialist involvement as required, throughout their regular contact. The authors speak largely from their experience in the US healthcare model, and it is important to acknowledge the variability in primary care infrastructure across global settings.
